# A pilot randomized clinical trial of a smartphone-based application to support at-home PSA screening and culturally tailored prostate cancer education for African American men: A study protocol

**DOI:** 10.1016/j.cct.2024.107737

**Published:** 2024-11-10

**Authors:** Jordan Neil, Bingjing Mao, Ruosi Shao, Motolani E. Ogunsanya, Summer Frank-Pearce, Michael Businelle, Michael Cookson, Kelly Stratton, Mark Doescher, Stephanie Pharr, Valerie Moise, Brianna Fleshman, Jack Fronheiser, Kimberly Estrada, Iván Flores, David Bradley, Ashley Kendrick, Adam C. Alexander

**Affiliations:** a TSET Health Promotion Research Center, Stephenson Cancer Center, the University of Oklahoma Health Science Centers, Oklahoma City, OK, USA; b Department of Family and Preventive Medicine, College of Medicine, University of Oklahoma Health Sciences Center, Oklahoma City, OK, USA; c School of Public Health, Louisiana State University Health Sciences Center - New Orleans, United States; d School of Communication, Florida State University, Florida, USA; e College of Public Health, University of Oklahoma Health Sciences Center, Oklahoma City, OK, USA; f Department of Urology, College of Medicine, University of Oklahoma Health Sciences Center, Oklahoma City, OK, USA; g Community Outreach and Engagement Core, Stephenson Cancer Center, the University of Oklahoma Health Science Centers, Oklahoma City, OK, USA; h School of Communication, Florida State University, Tallahassee, FL, USA

**Keywords:** Prostate cancer, Mobile health, Prostate-specific antigen, African American, cancer screening, Disparities

## Abstract

**Background:**

Prostate cancer is the most diagnosed cancer in Black/African American men (AA) and the second‑leading cause of cancer-related deaths. A prostate-specific antigen (PSA) blood test is an early detection screening tool for prostate cancer, but uptake of PSA screening remains low among AA men. Greater PSA screening rates among AA men, coupled with earlier treatment, may reduce disparities in prostate cancer outcomes, including mortality. The current pilot study will test the first-of-its-kind mobile health (mHealth) app to improve prostate cancer knowledge and increase PSA screening uptake among AA men using home-based screening methods.

**Methods:**

AA men aged 55 to 69 and are not up to date with PSA screening will be randomly assigned 1:1 to receive a prostate cancer screening app: Prevention Taskforce App (Taskforce App; control condition) or the Prostate Cancer Genius App (Genius App; intervention condition), which was developed specifically for AA men.

**Results:**

We will evaluate the preliminary efficacy of the apps via post-intervention group differences on the validated 18-item Prostate Cancer Knowledge Scale (primary outcome). We will also explore post-intervention group differences in perceived engagement, accessibility, and acceptability between the apps. Finally, we will derive preliminary estimates of PSA screening rates between study conditions and identify mechanisms of screening adherence.

**Discussion:**

mHealth apps offer promise to improve prostate cancer knowledge and screening rates among AA men. Demonstrating the preliminary efficacy of the Genius App will support future fully-powered mHealth interventions to address health disparities.

## 1. Introduction

Disparities in prostate cancer incidence and mortality for AA men have persisted for almost half a century and are among the most extreme disparities of all cancers in the United States (US) [1]. Compared to national estimates, AA men in Oklahoma are disproportionately affected by prostate cancer incidence (104.5 per 100 k vs. 165.3 per 100 k) and mortality (19.1 per 100 k vs. 42.8 per 100 k) [2]. The prostate-specific antigen (PSA) blood test is an early detection screening measure for prostate cancer [3,4]. Approximately 30 % of the variation in mortality rates from prostate cancer can be attributed to the accessibility of the PSA test [5], but AA men are less likely to have screened with a PSA test compared to non-Hispanic White men nationally [6] and in Oklahoma [7]. Greater PSA screening uptake among AA men may reduce the disparate mortality burden of this disease [8,9]. While concerns about overdiagnosis and overtreatment of low-risk tumors from PSA screening remain, AA men benefit most from screening as they exhibit higher PSA levels, earlier disease progression, and are more likely to harbor genomically aggressive prostate cancer [2,6]. As such, recent guidelines [10] now recommend PSA screening for AA men and acknowledge that existing guidelines were developed from clinical trials that included predominantly White men and, thus, did not reflect racial and ethnic disparities in prostate cancer incidence and mortality in the US [11].

Knowledge about prostate cancer and the potential benefits of screening remain low among AA men compared to other racial groups [12]. Further, the PSA test is often completed within clinics, limiting access to AA men without a registered primary care provider, health insurance, reliable transportation, or sufficient time with a busy work or family schedule [13–16]. Increasing prostate cancer knowledge and accessibility to the PSA test could potentially be an important intervention target to reduce prostate cancer disparities among AA men. Prostate cancer education and/or screening interventions for AA men are often designed to be delivered in community-based settings, such as churches and barbershops [17–22]. However, localized interventions targeted at specific segments of the AA population lack the reach and scalability needed to have a nationwide impact on prostate cancer disparities. With the ubiquity of smartphones [23], mobile health (mHealth) apps are an effective modality to deliver highly scalable and remote interventions for prostate cancer screening and early detection. A past systematic review identified commercially available apps to promote informed decision-making about prostate cancer screening [24]. However, none of the apps included sufficient information to meet the American Cancer Society’s Early Detection Guidelines [25] and most were inactive or outdated. Furthermore, most apps were not tested within a randomized clinical trial and thus present substantial methodological limitations when evaluating their potential efficacy. No apps have been explicitly designed to promote PSA screening among AA men (e.g., using culturally sensitive language and tailored imagery). Past studies have demonstrated that culturally tailored interventions are more effective at increasing patient knowledge and intentions to screen for cancer [26,27]. Yet, there remains an opportunity to develop mHealth interventions that combine tailored information for AA men (i. e., about their greater risk for prostate cancer) with strategies that increase the accessibility of PSA screening kits.

At-home cancer screening tests have been used extensively for other cancers (e.g., fecal immunochemical tests for colorectal cancer) [28,29], and within AA communities [30–32], but at-home screening for prostate cancer has received limited investigation [33]. No existing apps integrate the ability to order an at-home PSA test and provide step-by-step navigation to complete the test. Therefore, this pilot project will test the preliminary efficacy of a prostate cancer screening app (Genius App) designed to improve prostate cancer knowledge and provide tailored screening navigation for AA men in Oklahoma. The Genius App includes several culturally responsive features to overcome limitations present in existing prostate cancer screening apps and sustain engagement. Such features include culturally tailored language and medical illustrations of AA men, testimonial videos from AA survivors, and the ability to schedule a call with a survivor from the local community to discuss prostate cancer screening and receive social support. The Genius App capitalizes on momentary changes in motivation to screen by offering on-demand ordering of at-home PSA test kits, a novel approach that could significantly impact screening rates among AA men in Oklahoma.

## 2. Method

AA men (*N* = 80), aged between 55 and 69, and who are not up to date with PSA screening according to recommended United States Preventive Services Taskforce (USPSTF) guidelines [17], will be randomly assigned 1:1 to one of two study conditions: the Taskforce App (control condition) or the Genius App (intervention condition). The Taskforce App [34] condition includes an educational app previously developed by USPSTF and the U.S. Department of Health & Human Services, a study app that includes study assessments, and a button to order an at-home PSA test. All participants will receive a study-provided, pre-paid smartphone (Samsung Galaxy S21). Both conditions will complete a baseline survey, use their respective smartphone app for 30 days, complete four once-weekly smartphone surveys, complete a one-month post-intervention survey, and be asked to complete a semi-structured exit interview after their post-intervention survey. The Genius App (see Fig. 1 in Supplemental Materials for app comparisons) will use responses to weekly smartphone surveys to guide tailored navigation content for the at-home PSA test. The study launched in February 2023 during Black History Month and is currently enrolling participants. The University of Oklahoma Health Sciences Center (reference number: 14666) institutional review board approved the protocol presented in this study. This trial was registered at ClinicalTrials.gov (NCT05331638).

### 2.1. Inclusion/Exclusion criteria

Individuals will be included in the study if they: (1) live in Oklahoma, verified via a driving license or addressed mail; (2) self-identify as “male” and “Black or African American”; (3) self-report no previous diagnosis of prostate cancer; (4) self-report have not completed a PSA test or digital rectal exam (DRE) within the past two years; and (5) self-report are between the ages of 55 and 69. Age criteria were restricted to this range to match the USPSTF guidelines and information in the Taskforce App.

### 2.2. Study recruitment and procedure

Participants will be recruited using flyers posted in predominately AA organizations (e.g., barbershops and churches), digital flyers posted on social media platforms (e.g., Facebook, Instagram, and Twitter), and via an online clinical trial recruitment company (BuildClinical). Those who scan the QR code on recruitment flyers or click the link on social media platforms will complete a brief online screener survey using the web-based Research Electronic Data Capture [35] (REDCap) platform to determine study eligibility. REDCap is a secure, HIPAA-compliant web-based application designed for data collection and consenting participants for research studies. Research study staff will contact adults who qualify for the study and schedule a phone consultation to discuss study participation. To reduce the likelihood of fraudulent enrollments in the study, participants must provide a picture of their valid driver’s license or mail with their name and proof of address.

Those deemed eligible and willing to participate during the enrollment phone call will be provided with information about the study’s purpose, goals, and procedures and asked to electronically sign a consent form and a HIPAA 1 form. To do so, participants ‘sign’ a digital version of both forms in REDCap. To do so, participants will receive a unique REDCap link and sign an e-consent instrument for the IRB-approved document. After completing the enrollment call, the participant will be granted access to their REDCap baseline survey, which can be completed on any internet-connected electronic device. After completing the baseline survey, the research team will mail a welcome packet, physical copies of the signed study forms, and a pre-paid smartphone (Samsung Galaxy S21), with the INSIGHT^™^ app downloaded onto the phone, to the participant’s home address. All participants are required to use a study-provided smartphone.

Once the participant receives the phone, they will be instructed to complete an activation assessment in the INSIGHT^™^ app. This activation assessment determines if the participant can fully utilize the app’s capabilities. Participants who pass the activation assessment are randomly assigned to receive either the Taskforce App or the Genius App conditions. Access to both apps is granted using a unique code texted by the research team to the participant. Once participants enter the unique code on the home screen of the INSIGHT^™^ app, they will gain access to the features available in their study app (i.e., Taskforce App or Genius App). Participants assigned to the Taskforce App condition are required to download the educational app and are provided extra instructions on how to do so from the Google Play Store. Once downloaded, the educational app is easy to use and navigate. Participants enter their height, weight, age, sex, tobacco use, and sexual activity history and are given screening recommendations based on those characteristics. Due to the study’s eligibility criteria, participants will receive access to prostate cancer screening information. The research team will schedule a second phone call with participants after they complete the activation assessment to ensure they entered their unique code correctly and have access to their study app content and surveys. Participants who do not pass the activation assessment will be withdrawn from the study prior to randomization and required to return the study smartphone via a return mailing package. Upon entering a correct study code, participants will use their assigned app(s) for 30 days and complete a weekly survey for four weeks [36] during the study period. After using the app for 30 days, participants will be granted access to their REDCap post-intervention survey and asked to schedule their exit interview with a research team member. Once the REDCap post-intervention survey and exit interview have been completed, the participant will return the study materials to the research team using a study-provided return mailing package (see Fig. 2 in Supplemental Materials for participant flow).

### 2.3. Study randomization

Participants (*N* = 80) will be assigned using stratified randomization to one of two study conditions: the Taskforce App (*n* = 40) or the Genius App (n = 40). Participants will be stratified on family history of prostate cancer (yes/no) and reading level (> 8th grade vs. ≤ 8th grade; based on scores on the Rapid Estimate of Adult Literacy in Medicine – Short Form [37]) to ensure that both study conditions are balanced on important factors associated with prostate cancer knowledge and screening [38–41]. An online baseline survey will be administered before randomization, and a post-intervention survey will be administered immediately after the intervention.

### 2.4. Mobile health platform

The INSIGHT^™^ Mobile Health Platform, developed by the mHealth Technology Shared Resource at the NCI-designated Stephenson Cancer Center [42], enables researchers to deploy technology-based assessments and interventions. Participants will use a study-provided smartphone (Samsung Galaxy S21) with cellular service to access study apps and complete all study activities.

### 2.5. Study conditions and app features

See Table 1 for more details about the features included in the study apps. Below is a brief description of each app and its associated features.

#### 2.5.1. Taskforce app

The Taskforce App is free on Google Play and Apple App Stores [17]. It is a practical resource designed to help health professionals and patients identify and prioritize appropriate screening based on USPSTF recommendations. The app provides screening recommendations based on characteristics input by the user and provides generic information about prostate cancer and screening.

#### 2.5.2. On-demand app features

In addition to using the TaskForce app, participants assigned to this study condition use a companion app housed on the INSIGHT^™^ mHealth Platform. On the home screen of their companion app, the following menu options can be assessed at any time during study participation by clicking on their respective button on the home menu.

##### 2.5.2.1. What is the US prevention taskforce app.

This feature provides an overview of the TaskForce App and instructions for accessing it on their study phone.

##### 2.5.2.2. Order a home-based PSA test kit.

Participants can order an imaware^™^ PSA test kit from their mailing address and navigate to register for health insurance if they are currently uninsured.

##### 2.5.2.3. Report my PSA test result.

Participants can report the results from the imaware^™^ PSA test kit PSA test or if they choose to go to a provider and complete a clinic-based PSA test.

##### 2.5.2.4. Track my rewards.

Participants can track the compensation they have earned after completing study assessments (see Compensation section).

##### 2.5.2.5. Report a problem.

Participants can report any problems encountered during study participation via an encrypted email sent directly to the research team, who typically respond within 24 h during business days (Mon-Fri).

#### 2.5.3. Prostate cancer genius app

The design of the Genius App is informed by our preliminary studies and established theoretical frameworks. The Genius App includes the following on-demand features:

##### 2.5.3.1. Read the prostate cancer e-book.

Participants have access to eight learning modules created specifically for AA men. A Black literacy expert-reviewed content in the modules to ensure the language was culturally tailored and medical illustrations of AA men were created to tailor the imagery. These learning modules cover various topics on prostate cancer, risk factors, screening, etc., followed by an interactive quiz that encompasses questions about the content (see Table 2 for an overview). Two new learning modules become available on Days 7, 14, 21, and 28. Modules are accessible any time after their scheduled study day release.

##### 2.5.3.2. Watch videos about prostate cancer.

Participants can select from various short video testimonials (1–10 min) from four AA prostate cancer survivors living in Oklahoma. These videos are narrative-focused and convey powerful, personal stories about key topics of a prostate cancer journey for AA men (see Table 3) [43].

##### 2.5.3.3. Talk with a prostate cancer survivor.

The survivors who tell their stories in the videos also provide social support to participants in the study as “peer coaches.” [44] Participants can request a conversation with one of the four survivors and also select from a list of topics to prioritize (see Table 3). The study team schedules these conversations, and survivors use a study phone.

Similar to the Taskforce App, the Genius App also includes the following On-Demand features: (1) Order a Home-Based PSA Test Kit, (2) Report My PSA Test Results, (3) Track My Rewards, and (4) Report a Problem.

#### 2.5.4. Scheduled assessments

Both study apps use on-demand features and scheduled assessments to promote engagement with app features. For example, the Genius App has a scheduled assessment on Day 2 of study participation (“Prostate Cancer Survivor Introduction”) to deliver a video introducing the names and backgrounds of the participating prostate cancer survivors. However, participants can complete an on-demand feature (“Talk With a Prostate Cancer Survivor”) at any time during the study to schedule a phone conversation with one of the four prostate cancer survivors (see Table 4 for an overview of scheduled assessments).

### 2.6. Theoretical framework

**Fig. 1.**
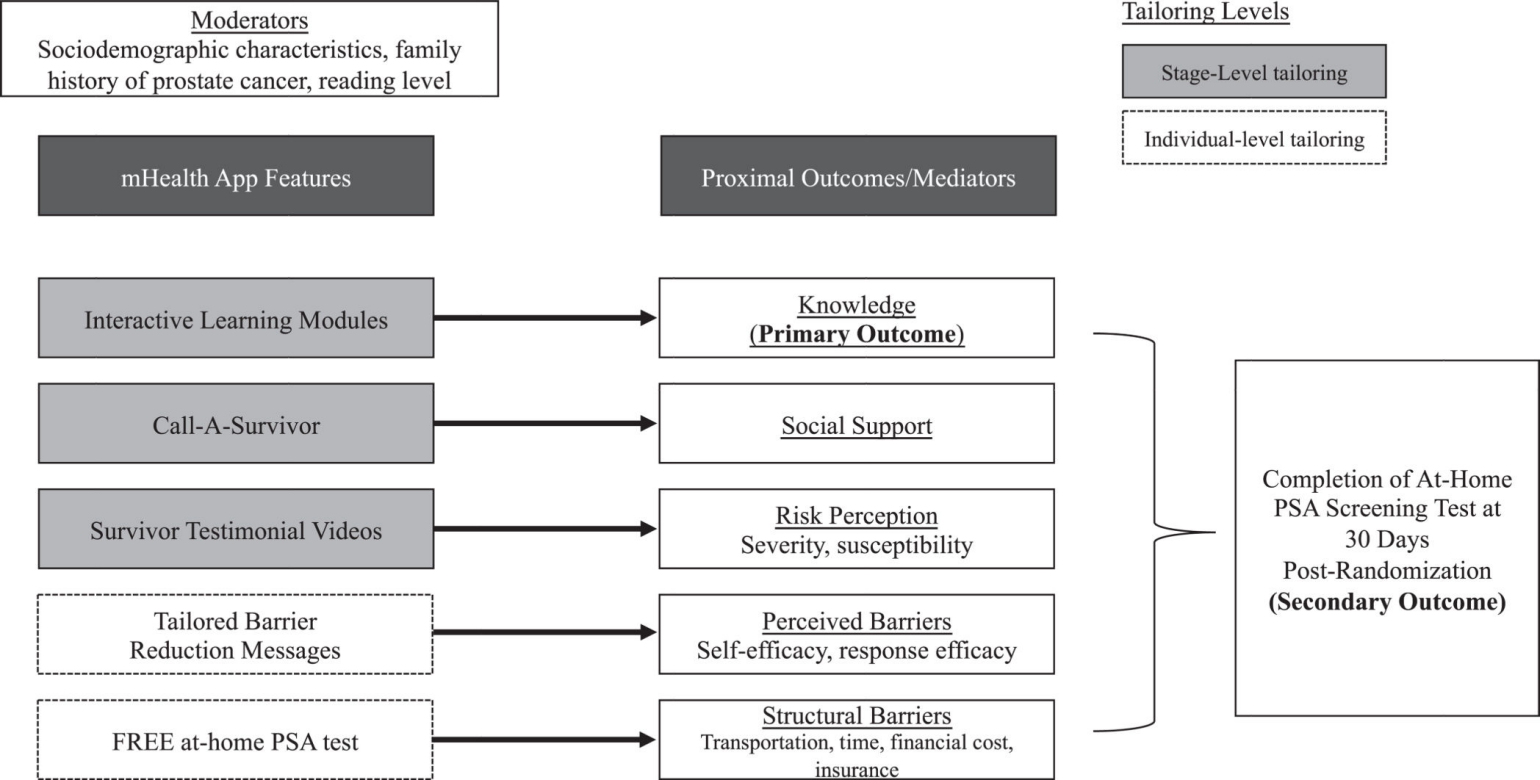
Conceptual diagram for Prostate Cancer Genius App.

Each unique app feature and assessment within the Genius App is posited to affect a proximal outcome, such as prostate cancer knowledge, which in turn is believed to act as a mechanism through which the app feature will increase the completion of PSA screening (see Fig. 1 for conceptual diagram). For example, the Health Belief Model (HBM) posits that knowledge about a disease is an antecedent to motivated information processing. Thus, we have included culturally tailored interactive learning modules and testimonial videos to improve knowledge about prostate cancer, risk factors, screening, etc. Similarly, the HBM posits that people will engage in a health behavior when they acknowledge the high perceived severity of a health threat, their high susceptibility to that threat, and the benefits of the health behavior to remove the threat (i.e., PSA screening). The survivor testimonial videos were filmed and construct-mapped to address these pathways. The HBM highlights the need to partner interventions that address both perceived and structural barriers to engaging in health behavior. Thus, we provide access to on-demand screening through a free at-home PSA test [45,46] and employ the Extended Parallel Process Model (EPPM) to construct risk and efficacy messaging through the tailored barrier reduction messages [47,48]. Social support is considered a third protective mechanism when coping with health threats [49–51]. Social support can directly promote better management of health threats and increase engagement with health behavior change. Social support buffers individuals against emotional burden when preparing to change or complete a health behavior, so we incorporated a feature that allows participants to contact and have conversations with prostate cancer survivors [49–51].

### 2.7. At-home PSA test kits

The study team partnered with imaware^™^ (https://www.imaware.health/) to deliver study-provided at-home PSA test kits to men who ordered one during the study. The at-home PSA test collects a small sample of blood using the finger prick, which correlates strongly with PSA levels in venous blood, and CLIA-certified, CAP-accredited labs analyze these kits, the gold standard in lab testing quality regulation [52–54]. Each kit has detailed instructions for how to do the finger prick correctly, and a video link will be provided to participants who need additional instructions. Participants can also request help from study staff to provide support on how to complete the test over the phone. Participants will mail their test kits to imaware^™^, and the company will analyze the blood sample. Once imaware^™^ analyzes the sample, the PSA results will be shared with the participant through a portal login (see Fig. 3 in Supplemental Materials).

### 2.8. Study surveys

#### 2.8.1. Screening survey

Participants will complete a REDCap screening survey when they click an advertisement for the study. The screener consists of 37 items that assess demographics, English literacy, prostate cancer and screening history, and other eligibility criteria. See Table 5 for a list of variable categories across assessment types.

#### 2.8.2. Enrollment assessment

Participants who are found to be eligible for the screener will be contacted by phone and will complete the final screening items. Specifically, participants will e-sign study documents, including informed consent, a HIPAA form to enable contact with imaware^™^, and an agreement to take care of and return the study phone. Participants will be asked about their preferred assessment time for weekly smartphone-based assessments. In addition, participants’ literacy will be assessed using the seven-item Rapid Estimate of Adult Literacy in Medicine – Short Form [37].

#### 2.8.3. Baseline survey

Participants will complete the REDCap baseline survey before being randomly assigned to a study condition. The baseline survey consists of a total of 365 items that assess sociodemographic characteristics (e.g., education, insurance status), social determinants of health (e.g., food and financial security), physical (e.g., Behavioral Risk Factor Surveillance System health conditions) and psychological wellbeing (e.g., GAD-7, PHQ-9), health behaviors (e.g., smoking, sleep), patient-provider relationship (e.g., medical mistrust), general cancer screening history and attitudes (e.g., screening self-efficacy, perceived threat), as well as prostate cancer-specific knowledge and attitudes (e.g., decisional conflict scale).

#### 2.8.4. Weekly smartphone-based surveys

Using the INSIGHT^™^ mHealth Platform, participants in both study conditions will be prompted during their preferred assessment time by their smartphones to complete weekly surveys for four weeks (on days 7, 14, 21, and 28 post-randomization). The phone will audibly and visually cue these surveys for 30 s. The assessment will be recorded as missed if a participant does not respond after five prompts. All assessments will be date- and time-stamped for future analyses. Weekly surveys assess the frequency of app use, satisfaction with assigned app content, knowledge and perceptions of prostate cancer screening, readiness to complete a PSA test, and current emotional states.

#### 2.8.5. Post-intervention survey

Participants will gain access to a post-intervention survey 30 days after they are randomized to a study group. The post-intervention survey assesses prostate cancer health and screening beliefs, attitudes, behaviors, and acceptability with the smartphone app (e.g., user engagement scale, system usability scale).

#### 2.8.6. Exit interview

All participants will complete the qualitative exit interview that measures their overall attitudes toward the study and the prostate cancer screening process. Participants assigned to use the Genius App will be asked about their experience completing learning modules, their thoughts about survivor videos, and their conversations with survivors. All participants will be asked about the efficacy of using the app to order an at-home PSA test kit and attitudinal changes in prostate cancer screening after using their respective app. See Table 6 for qualitative domains asked by study condition.

### 2.9. Study compensation

All participants who enroll in the study will receive a Greenphire Mastercard [55,56] to facilitate payment for completing study surveys. Participants earn $25 for completing the enrollment survey, $50 for completing the baseline survey, $50 for completing the post-intervention survey, $50 for completing their exit interview, and $25 for returning their study materials after completing all study activities. Participants can earn an extra $25 for completing their baseline and post-intervention survey within 72 h of being granted access to them. Participants can earn $20 for each weekly survey they complete on their smartphones (up to $80 in total compensation). Participants can earn up to $330 on their Greenphire Mastercard if they complete all study activities.

### 2.10. Outcome measures

#### 2.10.1. Primary outcome

The primary outcome measure is prostate cancer knowledge, assessed using the Prostate Cancer Knowledge Scale [57] administered at baseline and post-intervention surveys. The scale consists of 18 items that assess screening (6 items), risk factors (5 items), and warning signs (7 items) of prostate cancer. This scale has strong internal consistency (α = 0.80) and has been validated for AA adults. [57]

#### 2.10.2. Secondary outcomes

Imaware^™^ will share information about PSA results with our research team to allow for the calculation of test completion rates. Participants have 30 days to order a PSA test kit. Perceptions of how engaging the app will be measured during the post-intervention assessment using the User Engagement Scale (UES) [58], which is a 31-item multidimensional scale measuring six distinct dimensions of (a) aesthetic appeal, (b) focused attention, (c) novelty, (d) perceived usability, (e) felt involvement and (f) endurability. The System Usability Scale (SUS) [59] will measure *perceived accessibility*, a 10-item bidimensional scale of (a) how useable and (b) how learnable the app was.

### 2.11. Power analysis

We anticipate that 20 % of the full sample will be lost to attrition; therefore, the sample size power calculation is based on 60 participants. Assuming the mean (standard deviation) of baseline knowledge in the Taskforce App is 11.1 (3.1) [60] and a two-sided alpha of 0.05, we will have 80 % power with *N* = 60 (30/group) to detect a difference of 2.3 points on Prostate Cancer Knowledge Scale.

### 2.12. Analytical plan

We will assess pre-post intervention group differences on the Prostate Cancer Knowledge Scale using a two-sample *t*-test. Linear regression will also be used to estimate the post-intervention difference on the Prostate Cancer Knowledge Scale while accounting for the participant’s baseline score on the Prostate Cancer Knowledge Scale and demographic and psychosocial characteristics (e.g., age, insurance status). We anticipate that the participant characteristics, family history of prostate cancer, and literacy level will be well balanced by randomization. However, we will examine the potential moderating effect of these variables on pre-post group differences in the Prostate Cancer Knowledge Scale.

PSA screening test rates will be calculated for (1) the overall screening rate across both groups and (2) Taskforce App vs. Genius App. A chi-square test of independence (or Fisher’s exact test, as appropriate) will be used to compare PSA screening test completion rates between study conditions. We will also examine between-group differences (Taskforce App vs. Genius App) at follow-up through the overall score of UES and each of the six dimensions using two-sample *t*-tests. For perceived accessibility, we will also use two-sample t-tests to explore between-group differences, as measured via the overall score of SUS.

#### 2.12.1. Qualitative data analysis

Recordings from exit interviews will be transcribed and then loaded into NVivo [61] (Version 12) for analysis. An inductive thematic approach will inform the qualitative analysis [62,63]. The investigative team (led by Dr. Neil) will create the codebook and then code two interviews as a team, which will be used to revise the codebook (if needed). Once consensus has been reached on coding, the coded transcripts will be reevaluated, and themes will be identified within and across codes. These themes will highlight the study apps’ strengths and weaknesses, providing insight into improving acceptability and the participant experience.

## 3. Results

Data collection is expected to finish in September 2024, and primary and secondary data are expected to be analyzed in Fall 2024, with papers on these anticipated data starting in late 2024 and early 2025.

## 4. Discussion

Prostate cancer is the most diagnosed cancer in AA men and the second‑leading cause of cancer-related deaths [64–66]. The current pilot study will test the first-of-its-kind mHealth app to improve prostate cancer knowledge and increase PSA screening uptake among AA men using home-based screening methods. The Genius App is theory-driven and community-guided, offering culturally tailored education about prostate cancer for AA men. Our primary hypothesis is that participants randomly assigned to the Genius App will report significantly greater knowledge on the Prostate Cancer Knowledge Scale post-intervention than participants randomly assigned to the Taskforce App. We will also derive preliminary estimates of the effects of each prostate cancer screening app on at-home PSA screening test completion rates.

This study has many notable strengths. First, it focuses on a high-risk group with a limited history of prostate cancer screening, concentrating on an important public health disparity [64]. Second, it addresses many of the methodological limitations of existing prostate cancer screening app pilot studies through a rigorous experimental design [24,67]. Third, via weekly smartphone assessments, the study will measure mechanisms of behavior change, including the cognitive and emotional changes that individuals go through when engaging with a longitudinal cancer screening intervention that is otherwise not collected in standard screening interventions. Fourth, the remotely delivered, culturally responsive intervention addresses both patient-level factors (i.e., equitable access to screening) and structural-level factors (i.e., equitable access to culturally tailored information for AA men) that disproportionately affect AA men [68–71].

As with any study, however, there are some limitations. First, the investigative team rigorously controls access to the app to better understand its use during the pilot study. Multiple steps are required (e.g., verbal consent, signing a HIPAA waiver, and completing an activation assessment to ensure the app is downloaded correctly), and all participants must use a study-provided phone. Smartphone ownership is high across socioeconomic status, but the quality of smartphones varies greatly [23]. To ensure access to the app is equitable and functions correctly, study phone use was deemed necessary. Future studies that test the app will not require a study phone unless participants request a phone. Second, although the weekly smartphone assessments offer an important theoretical contribution by exploring mechanisms of behavior change, they may have a small intervention effect by reminding participants to screen in the control group. Despite these limitations, a successful demonstration of the preliminary efficacy of the Genius App will support a statewide effectiveness trial and integration across the OU Health medical system.

## Supplementary Material

MMC1

## Figures and Tables

**Table 1:** Overview of App Features by Study Condition.

App Features	Prostate Cancer Genius App	Taskforce App
*App Instructions*	X	X
*USPSTF Companion App*		
Generic information about prostate cancer and screening	–	X
*Read the Prostate Cancer e-Book*		
Culturally tailored information about prostate cancer and screening.	X	–
*Watch Videos about Prostate Cancer*		
Experience sharing from AA prostate cancer survivors.	X	–
*Talk with a Prostate Cancer Survivor*		
Schedule a call and select a topic to talk with a local AA prostate cancer survivor.	X	–
*Order a Home-Based PSA Test Kit*		
imaware^™^ PSA test kit mailed to home with return mailer.	X	X
*Report My PSA Test Results*		
Participants can report imaware^™^ or a clinic-based PSA test result completed during the study.	X	X
*Track My Rewards*		
Displays the amount of compensation received for completed study assessments.	X	X
*Report a Problem*		
Email the study team if the app isn’t functioning	X	X

**Table 2:** Overview of learning modules in the Prostate Cancer Genius app.

Week*	Module	Educational Content
1	1 2	Introducing prostate cancer The risk of prostate cancer for the AA community
2	3 4	Screening methods and recommendations for prostate cancer How to communicate with healthcare providers about prostate cancer screening
3	5 6	How to complete the at-home PSA test Prostate biopsy exam and results interpretation
4	7 8	How to seek social support from friends and family; The power of faith Masculinity and manhood in making decisions on prostate cancer screening

* *Note.* Two learning modules become available each week, and once presented, they can be accessed on demand.

**Table 3:** Overview of topics for testimonial videos and conversations with prostate cancer survivors.

Watch Videos about Prostate Cancer Topics	Talk With A Prostate Cancer Survivor Topics*
1. AA men who have survived prostate cancer.	1. Family history and warning signs of prostate cancer.
2. How to make faith-based decisions about prostate cancer.	2. Taking the PSA test or the DRE exam.
3. Does getting prostate cancer change your definition of manhood and masculinity.	3. Talking with the doctor about prostate cancer.
4. Knowing the signs and symptoms of prostate cancer.	4. Getting a prostate biopsy.
5. How friends, loved ones, and your community can support your health decisions.	5. Deciding which type of treatment is best for prostate cancer.
6. How you can take action to detect cancer early.	6. What it is like to be a prostate cancer survivor?
7. The benefits and costs of prostate cancer treatment.	7. How does prostate cancer affect your family and friends? 8. How has your life been affected by the side effects of treatment? 9. How has prostate cancer affected you mentally? 10. How has prostate cancer affected you spiritually? 11. Family history and warning signs of prostate cancer.

* *Note.* Participants select from a list of topics they want to speak to a survivor about. The survivor is informed of the participant’s preferred topic before they make the call. Multiple topics can be selected.

**Table 4:** Overview of scheduled assessment and menu options available in both study apps.

Name of Assessment	Brief Description	Assessment Type	Study Condition		Time Window
			TaskForce App	Genuius App	
Welcome Message	An assessment that provides an overview of the assigned study app and its associated features.	Scheduled Assessment^a^	X	X	Day 1 at the preferred assessment time.
Closing Message	An assessment that outlines the remaining steps needed to finish study participation	Scheduled assessment^a^	X	X	Day 30 at the preferred assessment time
Weekly Smartphone Assessment	This is a weekly assessment that assesses the frequency of app use, satisfaction with assigned app content, knowledge and perceptions of prostate cancer screening, readiness to complete a PSA test, and current emotional states.	Scheduled assessment^a^	X	X	Days 7, 14, 21, and 28 at the preferred assessment time
Home-Based PSA Test Reflection Assessment	This assessment measures the thoughts and feelings participants are experiencing while waiting for their results.	Scheduled Assessment^a^	X	X	Triggered 48 h after ordering a PSA test kit. Sent once per day at preferred assessment time until fully completed.
Prostate Cancer Survivor Introduction	An assessment that provides a brief introduction for all the prostate cancer survivors who provided testimonial videos and are participating in the app as “health coaches.”	Scheduled Assessment^a^	–	X	Day 2 at the preferred assessment time
Call A Survivor Check-In	An assessment that provides an opportunity for participants to schedule a conversation with a prostate cancer survivor.	Scheduled Assessment^a^	–	X	Days 5, 12, 19, and 26 at the preferred assessment time
Home-based PSA-Test Check-In	An assessment that provides an opportunity for participants to order a home-based PSA test.	Scheduled Assessment^a^	–	X	Days 3, 10, 17, and 20 at the preferred assessment time
Learning Modules	An assessment that provides an opportunity for participants to complete a learning module on prostate cancer.	Scheduled Assessment^a^	–	X	Days 2, 4, 9,11, 16, 18, 23, and 25 one hour after wake time

a A scheduled Assessment is triggered by the study-provided smartphone. The phone will audibly and visually cue these surveys for 30 s. If a participant does not respond after five prompts, the assessment will be recorded as missed.

**Table 5:** Overview of Domains Across Each Assessment Type.

Descriptive variable	Items	Full Bettery Of Assessments					
		Screener	Enrollment	Baseline	Weekly Smartphone	Post-intervention	Exit Interview
Screening	37	x					
Enrollment [1]	40		x				
Sociodemographic	33			x			
Health Behavior, Mental Health, Physical Health, and Healthcare Provider [2–10]	78			x			
General Cancer Health and Screening [6,11,12]	46			x			
Prostate Cancer Health and Screening [13–18]	78			x			
Psychosocial Factors [4,19–25]	130			x			
Prostate Cancer Health and Screening weekly [13–18]	40				x		
Prostate Cancer Health and Screening Post-Intervention [6,11,13,14,17]	54					x	
Smartphone App Review Questionnaire [26,27]	82				x	x	
Exit Interview	76						x
**Total number of items**	578	37	40	365	39	136	76
**Total time needed to complete the assessment (minutes)**	225 min	10–20 min	30–45 min	60–90 Minutes	10 min per week	30 Minutes	60 min

**Table 6:** Overview Of Qualitative Domains Asked By Study Condition In Exit Interview.

Qualitative Domain	Prostate Cancer Genius App	Taskforce App
1. Overall impressions about study.	X	X
2. Problems encountered while in study or using study phone/app.	X	X
3. Evaluation of culturally tailored app features (e.g., Prostate Cancer e-Book).	X	
4. Reasons why did/didn’t order at-home PSA test kit.	X	X
5. Experience with completing at-home PSA test kit and follow-up navigation	X	X

## Data Availability

No data was used for the research described in the article.
