# Supplementary material for: A pilot randomized clinical trial of a smartphone-based application to support at-home PSA screening and culturally tailored prostate cancer education for African American men: A study protocol
Source: Contemp Clin Trials. Author manuscript; Available in PMC 2024 Dec 9. (PMC11627590; doi:10.1016/j.cct.2024.107737)
Supplement: MMC1 [file NIHMS2036051-supplement-MMC1.docx]

**Supplemental Materials: Figures**


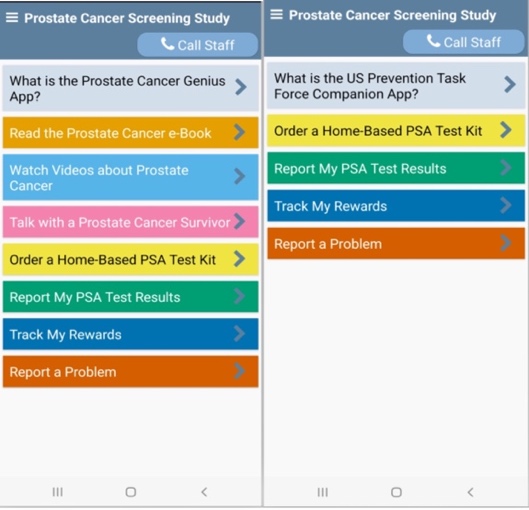


**Supplemental Figure 1**. Home screens for Genius App and TaskForce App

**Supplemental Figure 2.** Participant flow.


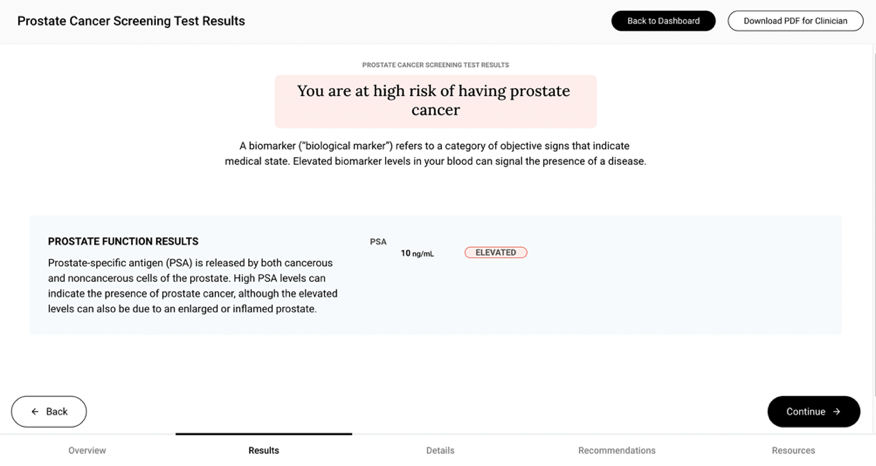


**Supplemental Figure 3.** Example of imaware^TM^ results communicated to participant.
